# Understanding and evaluation of the concept of “palliative psychiatry” among service users with chronic and treatment-resistant depression: a qualitative study of service user perspectives

**DOI:** 10.1186/s12888-026-08188-6

**Published:** 2026-05-21

**Authors:** Astrid Gieselmann, Sarah Potthoff, Robin Cole, Christian Otte, Katja Wingenfeld

**Affiliations:** 1 Berlin Institute of Health at Charité – Universitätsmedizin Berlin, Charitéplatz 1, 10117 Berlin, Germany; 2 Department of Psychiatry and Psychotherapy, Charité – Universitätsmedizin Berlin, Campus Benjamin Franklin Hindenburgdamm 30, 12203 Berlin, Germany; 3 Institute for Ethics, History and Theory of Medicine, University of Münster, Münster, Germany; 4 German Center for Mental Health (DZPG), Partner Site Berlin-Potsdam, Berlin, Germany

**Keywords:** Palliative psychiatry, Treatment-resistant depression, Chronic depression, Quality of life, Qualitative research

## Abstract

**Background:**

The concept of “palliative psychiatry”, which proposes a shift from curative goals to prioritizing quality of life for people with severe and persistent mental illness when remission seems unattainable, has been proposed to improve care for people with severe and persistent mental illness, yet service users’ perspectives, particularly in the context of depression, are underexplored. The objective of this study is to explore how individuals with chronic depression understand and evaluate a concept of “palliative psychiatry”.

**Methods:**

We conducted semi-structured interviews with 19 adults with chronic depression (age range 21–79 years; 63% women), many of whom had undergone intensive treatments such as electroconvulsive therapy or ketamine, recruited from three specialized wards in Berlin, Germany. Data were analyzed with thematic analysis, which is a systematic method for identifying, analyzing, and reporting patterns (themes) within qualitative data.

**Results:**

Thematic analysis revealed two main thematic patterns with several subcategories. The two main thematic patterns were “benefits and risks” as well as “needs and wishes”. Anticipated benefits included relief from perceived pressure to achieve remission and the avoidance of the psychological distress associated with repeated treatment failures. Risks included stigma linked to the term “palliative,” fear of premature transition away from potentially curative care, and loss of hope. Concrete service users’ needs and wishes included ongoing support without abandonment, care settings with a less institutional atmosphere, and transparent communication.

**Conclusions:**

Service users with chronic depression see benefits but also risks in a palliative orientation when curative goals appear unattainable, provided it does not imply therapeutic withdrawal. Potential future conceptual refinement of “palliative psychiatry” will require safeguards against premature categorization and careful terminology.

**Clinical trial number:**

Not applicable.

**Supplementary Information:**

The online version contains supplementary material available at 10.1186/s12888-026-08188-6.

## Background

Depressive disorders are among the most prevalent psychiatric conditions and up to 30% of patients follow a chronic, treatment-resistant course [1, 2]. Despite advances in pharmacological and psychotherapeutic treatments, a sizeable subgroup does not respond sufficiently to available interventions. Current estimates suggest that around 30% of patients with major depressive disorder meet criteria for treatment-resistant depression (TRD) [3, 4]. Within this group, in 10–20% of cases, remission is not achieved despite advanced interventions such as electroconvulsive therapy (ECT) or ketamine, sometimes referred to as “ultra-resistant” depression [3, 4]. Reported response rates for ECT and ketamine in TRD populations – typically 41% to 63% and 32% to 55%, respectively – further illustrate the limits of current interventions [5, 6]. When considering the overall population of individuals with depressive disorders, approximately 5–10% of individuals do not experience sufficient response even to these highly specialized treatments [3–5]. For these patients, standard therapeutic goals may no longer seem attainable, raising questions about alternative approaches to care.

In response to the limitations encountered when pursuing exclusively curative treatment goals, palliative care has developed as a complementary approach. Originally established within oncology, palliative care was primarily associated with end-of-life support in hospital settings. Over the past three decades, however, its scope has broadened considerably. Today, palliative care is increasingly provided in outpatient settings and across a wide range of serious, non-cancer conditions, often alongside disease-modifying or curative therapies [7, 8]. Its focus lies on alleviating suffering and improving quality of life [9, 10]. Within this development, the field of palliative care psychiatry has emerged at the intersection of psychiatry and palliative medicine, primarily focusing on the psychosocial dimensions of suffering in patients with advanced medical illness [11].

Building on the WHO definition of palliative care, the concept of “palliative psychiatry” has been proposed for individuals with severe and persistent mental illness (SPMI) when curative goals appear unattainable [12–14]. Similar to staging models in bipolar disorder and other mental illnesses, which propose that late or end stages may require “palliative-type approaches” rather than curative goals [15], “palliative psychiatry” seeks to explicitly acknowledge chronicity and prioritize quality of life. It aims to protect patients from overly burdensome interventions of limited benefit, while explicitly recognizing ongoing suffering and prioritizing quality-of-life concerns [16, 17].

A conceptual challenge in applying palliative care principles to psychiatry lies in the distinction between “curative” and “palliative” approaches. In contrast to somatic medicine, where disease and symptoms can often be separated, psychiatric symptoms largely constitute the disorder itself. Following Westermair et al., [14], we use the term “curative” in a broad sense to refer to treatment strategies primarily aimed at symptom reduction or remission, even if these do not address underlying causes or prevent recurrence. In contrast, a “palliative” orientation prioritizes quality of life and relief of suffering when remission appears unlikely.

Conceptual work further argues that with each failed attempt the benefit–harm ratio of remission-oriented treatment worsens, justifying a shift toward palliative goals in selected SPMI cases [14]. In this sense, futility has been proposed as a useful concept in psychiatry: when repeated interventions are highly unlikely to provide meaningful benefit, futility can serve as a signal to reorient care toward palliative principles rather than continued escalation [13, 16]. However, recent commentaries caution against an overly broad or premature application of the term “futility” in psychiatry, emphasizing that such labeling may risk undermining hope and neglecting still-available therapeutic options [18]. Conceptual analyses have further emphasized both the opportunities and limits of transferring palliative care principles into psychiatry [19]. Still, what exactly palliative psychiatry should entail, how it differs from existing models of chronic psychiatric care, and which patient groups it should apply to remains subjects of ongoing debate [20].

Recent case-based work has illustrated that palliative needs may arise across diagnostic groups. For example, a case report described the use of a palliative psychiatric approach for a patient with treatment-refractory catatonic schizophrenia after multiple therapeutic failures [21]. According to Trachsel et al., [13], palliative psychiatry has been suggested as potentially relevant for patients with treatment-refractory depression, severe chronic schizophrenia, or enduring anorexia nervosa, all of whom are at risk of either therapeutic neglect or overly burdensome interventions.

Empirical research on the concept of “palliative psychiatry” has so far focused primarily on healthcare professionals. A survey among psychiatrists in Switzerland found that 75% endorsed the view that palliative care could be beneficial for certain patients with severe mental disorders, as illustrated by specific case examples [22]. Similarly, a pilot study with psychiatric nurses suggested general support for integrating palliative principles into the care of individuals with SPMI [23]. While these findings point to a growing acceptance among professionals, the perspective of service users remains largely unexplored. A recent survey among U.S. psychiatrists showed that although most clinicians recognize the possibility that certain cases may be beyond the reach of curative treatment, many continue to offer interventions they personally regard as unlikely to be helpful [24]. This further highlights the need to examine how service users themselves understand the value and limits of ongoing treatment.

Against this background, service users’ voices are important for any future discussion or conceptual development of palliative psychiatry. However, to date no in-depth study has systematically explored how individuals with chronic depression perceive and evaluate this emerging concept. This study aims to fill that gap by examining how individuals with chronic and treatment-resistant depression – who are often confronted with the limits of current treatment options – understand and evaluate the concept of “palliative psychiatry”.

## Methods

We opted for realist thematic analysis because it allows for the identification of patterns of meaning (‘themes’) within participants’ accounts while staying close to their expressed experiences. This approach is particularly suitable for exploring under-researched topics where participants’ perspectives are not yet well understood [25]. This aligns with our study objectives which aim to investigate how people with chronic depression understand and evaluate the concept of palliative psychiatry. Our interdisciplinary research team included researchers with backgrounds in clinical psychiatry, psychology, medical ethics, sociology and international health sciences. Three team members have clinical experience in psychiatric wards specializing in depression, two as physicians (CO, AG), and one as a psychologist (KW). This diversity of perspectives and experiences within the research team was helpful for interpreting the data and ensuring intersubjective comprehensibility.

### Sampling method and participants

Nineteen service users with a history of chronic depression were selected from three specialized depression wards in a large psychiatric hospital in Berlin, Germany that is specialized in the treatment of chronic and treatment-resistant depression. Participants were eligible if they were at least 18 years old, had a history of multiple depressive episodes and were at a stable mental state at the time of the interviews, without acute severe depressive symptoms or suicidal ideation and were able to give informed consent. Participants were intentionally selected to ensure diversity across age, gender, socio-demographic background, and treatment history, in order to capture a broad range of perspectives on the concept under study. AG, who works as a physician in the clinic, approached service users and asked if they would be willing to participate in the study. Her clinical background may have facilitated participants’ willingness to share their experiences, but it may also have influenced how these experiences were articulated. This was considered during data analysis through regular team discussions. AG did not serve as the treating physician and made it explicit that participation could be declined without reprisal of any sort. The participants did not receive financial incentives.

Participants were 7 men and 12 women between the ages of 21 and 79 years (mean age 54.9). Participants represented diverse socio-demographic backgrounds and current life circumstances. Highest education varied from high school diploma to PhD.

All participants had long-standing and treatment-resistant depression and had undergone multiple therapeutic interventions, including pharmacotherapy, psychotherapy, and inpatient treatment. A substantial proportion had received intensive treatments such as electroconvulsive therapy and ketamine. Participant characteristics are summarized in Table 1.

**Table 1:** Participant characteristics

Characteristic	*n* (%)
**Total participants**	19 (100%)
**Age group (years)**	
20–39	2
40–59	9
≥ 60	8
**Gender**	
Female	12
Male	7
**Duration of illness**	
≥ 10 years	16 (84.2%)
**Treatment history**	
Pharmacotherapy	19 (100%)
Psychotherapy	19 (100%)
Electroconvulsive therapy (ECT)	8 (42.1%)
Ketamine treatment	8 (42.1%)
**Previous inpatient treatment**	19 (100%)

### Data collection

Face-to-face in-depth interviews were conducted between August 2023 and January 2024 in German language by AG. She conducted the interviews using a semi-structured interview guide that AG, SP, CO and KW had developed. A semi-structured format was chosen to allow for both consistency across interviews and flexibility to explore participants’ individual perspectives in depth. To provide background on the topic, participants were given a brief one-page summary outlining the central aspects of the concept of “palliative psychiatry”. The summary consisted of key points in a concise format and was explained verbally at the beginning of the interview. Participants were given the opportunity to ask clarifying questions to ensure a shared baseline understanding. The original summary was provided in German and has been translated into English for publication as supplementary material (Supplementary File 1). However, the participants were also asked what they thought palliative psychiatry should look like.

The interviews had two main sections: In the first part, interviewees were asked about their experience with chronic and treatment-resistant depression. In the second part, interviewees were asked about a hypothetical palliative psychiatry approach. This guide was designed to explore both their personal experiences and based on those experiences, hypothetical scenarios related to palliative psychiatry. In line with calls to focus on people with lived experience (PWLE) in shaping palliative psychiatry [26], the topic guide emphasized service users’ priorities, perceived benefits and risks, and preferences for care settings. Table 2 lists sample questions from the interview guide.

**Table 2:** Sample questions from the interview guide

Section	Questions
Initial questions	What experiences have you had with depression in your life? Can you describe difficult and easier episodes? What treatment approaches have you experienced, and how did you perceive their effectiveness?
Experience with treatment resistance	Have you experienced situations where curative strategies were not helpful? How did you feel about that? Which interventions may not have improved symptoms but nonetheless enhanced your quality of life? What needs do people with depression have when previous treatment attempts have failed?
Exploring a palliative approach in psychiatry	If we were to open a specialized unit for patients whose depression cannot be fully cured — what should such a unit look like? What should and should not be done there? What do you associate with the term “palliative” in psychiatry?
Closing questions	Is there anything important to you that we have not addressed?

AG carried out the interviews either in a research office or in the service user’s rooms on the ward. Interviews lasted from 33 to 89 min with an average of 51.6 min. The interviews were recorded, transcribed verbatim, pseudonymized and reviewed for accuracy. Additionally, sociodemographic and occupational data were collected for each participant, including age, gender and profession. We concluded data collection once we achieved a heterogenous sample according to our required characteristics and when no new themes emerged from additional interviews and thematic patterns were considered sufficiently developed.

### Data analysis

Data analysis was done using a realist thematic analysis approach. MAXQDA (version 2020.0.0.; VERBI GmbH), a qualitative coding software, was employed for data analysis. Two researchers (AG and SP) familiarized themselves with the data by repeatedly listening to the audiofiles and reading through the transcripts, to achieve immersion within the data. For the development of patterns of meaning, AG and SP coded a subset of initial interviews separately and identified an initial set of codes and emergent themes. The initial codes were revised, expanded and compared in regular data analysis meetings. Based on these discussions, AG and SP generated preliminary thematic patterns. AG and SP then coded the remaining transcripts using the existing codes and thematic patterns and adding further inductive codes as needed. Finally, AG and SP discussed and further developed the final thematic patterns, dividing them into several subcodes. To ensure intersubjective comprehensibility, AG presented these thematic patterns to the other study authors. Quotations were selected to illustrate each theme. Interview excerpts cited were translated into English after analysis with the help of a native speaker. In translating, we aimed to preserve the meaning of the complete utterance rather than the literal translation of individual words.

### Ethical considerations

The study was approved by the Ethics Committee of the Charité Universitätsmedizin Berlin. Participants provided written informed consent. Written and oral information was given to all participants before the interview started, with the opportunity to withdraw at any point. Participants received a detailed explanation of the procedures in place to maintain the confidentiality and protection of their personal data.

## Results

Participant characteristics are summarized in Table 1. The sample consisted of individuals with long-standing and treatment-resistant depression and extensive prior treatment experience.

Through thematic analysis, two overarching thematic patterns emerged, each characterized by several subcategories: (1) the risks and benefits of a palliative psychiatric approach, including specific prerequisites for implementing a palliative approach to minimize risks, and (2) the needs and wishes of service users with chronic depression.

### Theme 1: Risks and benefits of a palliative approach in psychiatry

#### Risks of a palliative approach

A prominent concern was the potential for stigmatization and exclusion of treatment-resistant patients. Interviewees spoke of a “dead end” and a “lost existence.” They were concerned about being “labeled” and viewed as “untreatable.” As one participant noted, *“Yes*,* because when you’re on your own*,* when you suspect it or when you know it for sure. Uh*,* that it doesn’t go away completely. So then you feel a stigma*,* that you’re somehow a bit of a leper to patients who haven’t had that experience yet. And then they ask*,* why not? And now I’m getting ECT [electroconvulsive therapy]*,* which is intense*,* and then these prejudices come up. That movie with Jack Nicholson is mentioned every single time.*” *(Interview participant – following IP – 13).*

Another significant risk we identified was the challenge of accurately determining when to shift from curative to palliative treatment. There was concern that an incorrect assessment could lead to a premature transition to palliative care, depriving a potentially “curable” patient of further therapeutic options. This was highlighted by a participant who stated, *“That*,* um*,* the situation of patients is misjudged and that they are deprived of opportunities to perhaps still get out and… That’s… That’s a real problem. That should be examined very carefully*,* very thoroughly.” (IP 13).*

Several participants voiced skepticism regarding the feasibility of integrating a palliative approach into the current healthcare system. Concerns were raised about structural limitations, such as financial barriers and staffing shortages, which could hinder the effective implementation of such a model. One participant stated, *“And (…) sometimes it’s good to slow down a bit*,* I think. But I believe that’s more due to the way hospitals are structured at the moment. You want to take care of people*,* but you know full well that you don’t have time and you have to write charts*,* and then you’re in the middle of a crisis*,* and I think a lot has to change there. But in itself*,* the concept is viable*,* but the conditions you need for it are going to be a tough fight*,* I think.” (IP 13)*.

The term “palliative” itself was perceived as problematic by some interviewees, who felt it was too closely associated with end-of-life care. This association was seen as potentially creating a sense of hopelessness or stigma. Participants frequently associated palliative care with end-of-life scenarios, particularly those involving incurable cancer. For this reason, the term “palliative” was often considered inappropriate for the concept. One participant stated, *“It sounds a bit harsh*,* like a kind of end-of-life care*,* like in cancer treatment. It’s not meant that way*,* but the thought does occur to you. When you hear that the patients are actually incurable and are only being accompanied.” (IP 1)*.

Several participants feared that introducing a palliative approach could leave patients feeling discouraged or deprived of hope. One interviewee noted, *“So for me personally*,* because even now*,* when I think I’m not cured or I’m not feeling much better*,* let’s say*,* I don’t want to give up. And if someone were to say to me now*,* well*,* you’re probably one of those 5% who would carry this with them their whole life or suffer from it*,* I think I would have a problem with that.” (IP 9).* Some participants felt that presenting a palliative option could lead to a perception of hopelessness so profound that it might heighten suicidal ideation.

#### Benefits of a palliative approach

Several subthemes show how a palliative approach could positively impact service user care in psychiatric settings. A significant number of interviewees acknowledged the existence of “palliative” cases within psychiatry—service users who have exhausted almost all available treatment options. Many participants identified with this category themselves or recognized others who fit this description. For example, one participant stated, *“Yes*,* I would definitely say so. Absolutely. And I would include myself in that group.” (IP 10)*.

Several participants found it helpful when the chronic nature of their condition was openly acknowledged and accepted by both themselves and their therapists. They emphasized the importance of a shared understanding that they would need to live with their illness, with clear communication from healthcare providers. One participant reflected, *“On the other hand*,* I think maybe I would accept it. And through this acceptance*,* it would be better.” (IP 10).* The acceptance of treatment-resistance was viewed as potentially reducing the psychological burden of striving for an unlikely full recovery. Interviewees reported that they found it beneficial when there was no pressure to achieve full remission.

Another benefit identified was the avoidance of “false hopes” associated with trying new treatments that might ultimately fail. A palliative approach was seen as a way to prevent the emotional toll of repeated treatment failures. Many participants described past experiences with unsuccessful treatment attempts as deeply frustrating and detrimental to their mental health. One participant described this cycle: *“Because in every phase*,* in every episode*,* things got better. Of course*,* you get your hopes up that things will really get better and stay better. And when that doesn’t happen*,* when things don’t turn out the way you imagined or hoped they would*,* you naturally fall back into a hole. And with every episode*,* that hole gets deeper and deeper and deeper.” (IP 3)*.

Some interviewees highlighted the potential cost savings associated with a palliative approach, noting that avoiding expensive, ineffective treatments could benefit the healthcare system. As one participant noted, *“It’s just that I think economically*,* this concept is certainly much more viable than constantly repeating costly procedures*,* where people are then somehow spat back into their lives because there are no alternatives. And then at some point they end up back here again. That’s my idea on the matter.” (IP 10)*.

### Theme 2: Needs and wishes of treatment-resistant service users

**Fig. 1:**
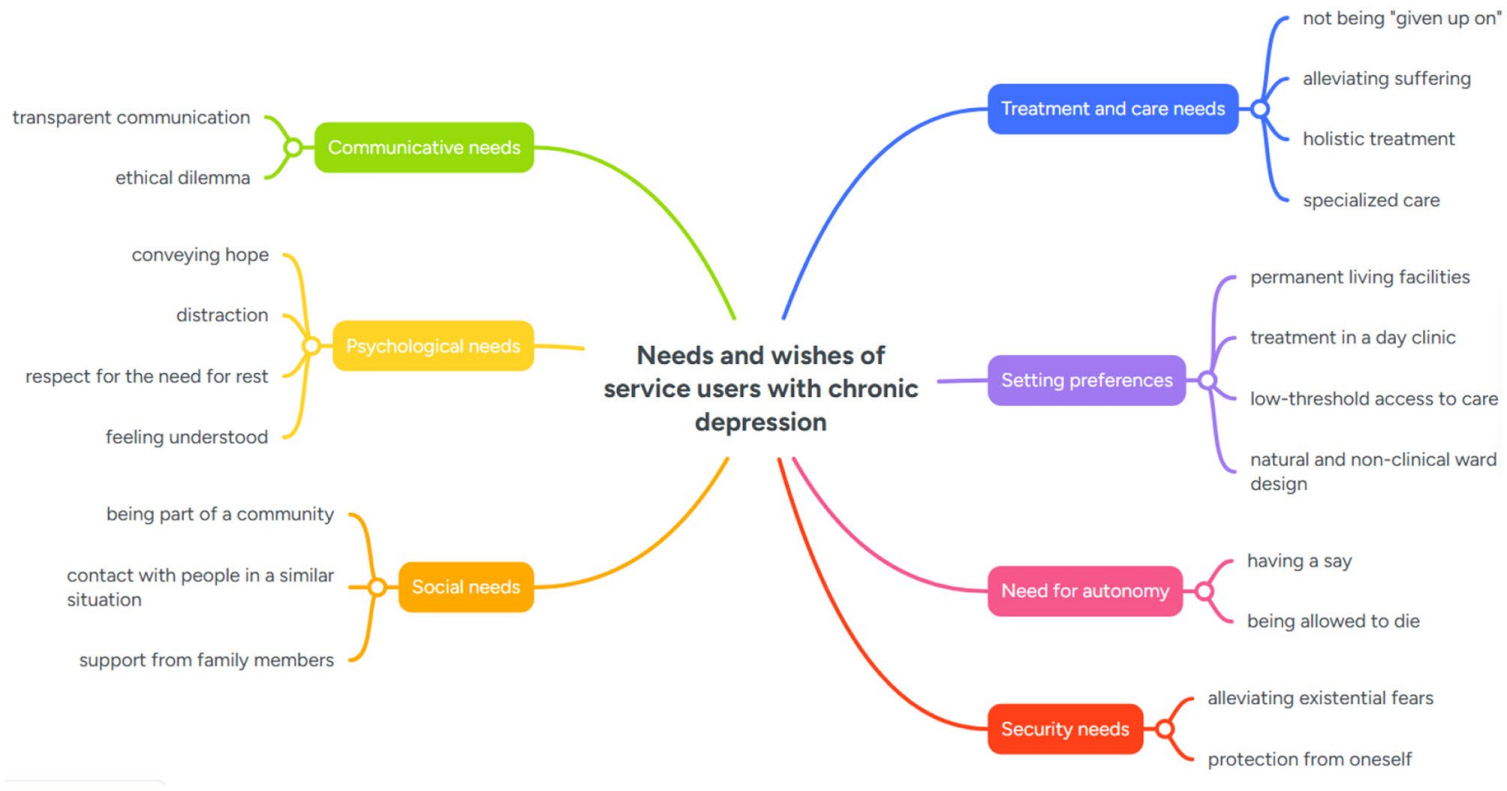
Needs and wishes of service users with chronic depression

Our analysis also showed specific needs of people with chronic depression who might be considered suitable for a palliative care approach. We categorized these needs into seven subcategories: treatment and care needs, setting preferences, psychological needs, social needs, communicative needs, autonomy and security. Figure 1 gives an overview of the subcategories and associated themes.

Our analysis also identified a range of needs and wishes expressed by participants in the context of discussing a potential palliative psychiatric approach. While the interview questions explicitly referred to such an approach, participants often drew on their broader experiences with depression and the mental healthcare system when articulating these needs. Accordingly, the themes presented here reflect both needs specifically relevant to a palliative orientation and more general care needs that participants perceived as insufficiently addressed in current psychiatric care.

#### Treatment and care needs

A central theme was the desire not to feel abandoned by the healthcare system, even when treatments had failed. One interviewee stressed, *“It’s very important that people don’t feel like they’re being abandoned.” (IP 13)* This reflects a fundamental need for ongoing support and the belief that care should persist despite the chronic nature of their condition.

Alleviating suffering emerged as a central priority for participants. Many expressed a need for symptomatic relief, even if only temporary. For instance, one participant explained, *“So tavor*,* or lorazepam*,* takes away*,* how should I put it*,* a piece of the suffering*,* a piece of the heaviness*,* of the emptiness.” (IP 11).* While acknowledging the potential risks associated with medications like benzodiazepines, many patients prioritized immediate relief over long-term concerns such as addiction.

Participants consistently called for individualized and holistic treatment approaches that consider their unique needs and broader life contexts. One participant stated, *“It really should be looked at holistically. So*,* where is the person*,* what is actually going on at home? Nobody comes home and checks to see what’s going on. And can you really get it sorted out?” (IP 2)*.

Lastly, participants highlighted the need for specialized care tailored to their unique challenges. This included the development of resources and expertise to address the specific needs of those with treatment-resistant depression. One participant suggested, *“I would like to see day clinics that are specially equipped for patients who are resistant to treatment.” (IP 11)*.

#### Setting preferences

Participants also expressed specific needs that service users with treatment-resistant depression would have regarding the setting and structure of their care environment.

Participants suggested that a ward setting might not be appropriate for a palliative care approach, particularly after an inpatient stay has ended but depressive symptoms persist. They expressed a preference for permanent living facilities or assisted living arrangements that provide ongoing support in a less clinical environment. One participant stated, *“I would prefer a facility where you can say that people can live here now… with a certain amount of protection. You could also go to assisted living. Something along those lines. In my opinion*,* a ward would also be a stay in a ward*,* which ends at some point. And chronic or treatment-resistant depression*,* or the systems that cause it*,* don’t end*,* they are persistent.” (IP 11)*.

The idea of a palliative setting in a day clinic or outpatient facility was also favored by many interviewees. This approach was seen as balancing support with maintaining everyday life. One participant explained, *“So actually*,* I think it should be structured more like a day clinic*,* because most people want to continue more or less a part of their normal lives*,* including friendships*,* etc.” (IP 1)*.

Participants expressed a need for low-threshold access to care, particularly given the low energy and capacity associated with severe depressive symptoms. One participant said, *“In any case*,* this person would like easier access to an appropriate specialist. Because at the moment*,* it can be really difficult and challenging. And when you feel so bad*,* then having to go out and search for help and sometimes struggle to find it*,* it’s a real challenge.” (IP 9)*.

Participants expressed a desire for a ward environment that feels more like everyday life, with a focus on natural and home-like elements, such as pets, plants, and non-hospital beds. One interviewee suggested, *“And apart from that*,* yes*,* I think architecture is also important*,* a kind of feel-good ward. Plants*,* perhaps*,* a therapy animal or a dog or a cat.” (IP 13)*.

#### Psychological needs

In addition to specific wishes regarding treatment, interviewees expressed various needs that we categorized as psychological needs of service users who are considered for a palliative approach.

A central psychological need was maintaining hope. Given the overwhelming nature of depression, participants often relied on their environment for encouragement. As one participant noted, *“But I think it’s also very important to give hope. Because*,* um*,* it’s not just older people who suffer from depression*,* but also many young people who still have their whole lives ahead of them. And when they’re told that it will never go away*,* I don’t know what goes through their minds.” (IP 3)*.

Participants also noted the importance of distraction from their symptoms as a way to manage their depression. Engaging in activities such as occupational therapy, structured daily routines, or physical exercise was seen as helpful in providing a temporary reprieve from depressive thoughts. One participant mentioned, *“It distracted me*,* because then I wasn’t thinking about anything*,* I was fully focused on this thing.” (IP 12)*.

Participants expressed a need for respect regarding their need for rest and the acknowledgment of their illness. They emphasized the importance of a calm treatment environment, free from excessive scheduling or pressure. One participant described, *“There should be no pressure*,* but it should be accepted that the person is ill and therefore cannot do much.” (IP 7)*.

Finally, patients expressed a need for understanding of their illness, both from healthcare providers and their personal support networks. Misunderstandings or lack of empathy from partners, family members, or friends was experienced as particularly stressful. One participant stated, *“Um*,* I would put understanding from my environment at the top of the list*,* because unfortunately it’s still the case. Our society still has a tendency to somehow dismiss things that aren’t visible*,* illnesses that aren’t visible. Yes*,* they are often taken lightly*,* even though the opposite is often true. Um. A lot of understanding.” (IP 15)*.

#### Social needs

Another strong theme was the desire to remain part of a community despite the illness. Participants expressed a preference for socializing with others who have shared experiences, which provided a sense of belonging without the pressure of having to explain or justify their condition. One participant shared, *“It is important that people with depression do not feel deficient. And that they also realize that they have resources and strengths. And that even people who are like this*,* as individual as they are*,* simply have a place in their illness. Where they can be*,* where they can live*,* as they are.” (IP 10)*.

Participants highlighted the benefit of maintaining contact with others who are experiencing similar challenges. Knowing that they were not alone in their struggles provided significant emotional support. One interviewee reflected, *“It was a valuable and pivotal experience for me to learn that there are people who feel the same way I do.” (IP 9)*.

Participants also described the desire to have support from family and friends. One participant said, *“It’s very important that the person doesn’t feel like they’re being abandoned. Family members should try to be there, even though it’s difficult. And friends, friends can try to get the person active.”* *(IP 13)*.

#### Communicative needs

Our analysis revealed significant communicative needs among service users with chronic depression, particularly concerning the topic of conveying prognosis. A key theme was the desire for transparent communication about the nature and progression of their condition. Participants emphasized the importance of explicitly acknowledging treatment resistance if that is the doctor’s belief. One participant said, *“I think it varies from person to person*,* because I’m someone*,* I want to know where I stand. Yes*,* and it would be harder for me if people were to more or less deceive me now. (…) That’s how it is*,* those are the facts. I’d rather have the facts*,* so that I know*,* than the other option*,* which would still trigger some hope that there might be some way out after all. But in the end*,* there isn’t.” (IP 11)*.

At the same time, we found that participants acknowledged the ethical complexities faced by clinicians in these conversations. Service users highlighted the critical role of hope in their journey. This tension between maintaining hope and delivering clear prognostic information was perceived as an ethical challenge for healthcare providers. One participant stated, *“I would say that’s the middle ground between not making false promises that you might not be able to keep*,* but also not discouraging people.” (IP 15)*.

#### Autonomy

In addition to that, our analysis showed that the theme of autonomy is a critical need for individuals with chronic depression. Participants expressed a strong desire to have a say in their treatment decisions, including which services they would like to accept and how long they need support from professionals. One participant stated, *“And maybe also a say in what they say we have on offer. What would they be interested in then?” (IP 7)*.

Particularly sensitive but important was the desire for the right to choose when to end their life, reflecting a need for self-determination, even to the extent of considering physician-assisted suicide in cases of unbearable suffering. One participant expressed, *“And now I have to say something very difficult. Why is this man not allowed to die? (…) Animals are given a mercy shot and also a bullet. Why shouldn’t a person who is only suffering be allowed to decide about their life every day and say*,* I don’t want to be a burden to anyone? (…) If someone has felt for years that they have lived long enough and every day of life is torture*,* why can’t they go? That’s the question*,* which I know is very challenging ethically*,* but I’ve asked myself that many times.” (IP 10)*.

#### Security

Our analysis showed that security needs were central and can be divided into two key areas. First, many participants described the profound impact that chronic depression can have on their ability to work, leading to situations where service users are at risk of financial insecurity. The desire for support in alleviating these existential fears was strongly expressed, with many participants advocating for palliative care settings that would address these issues and provide reassurance in the face of financial instability. One participant stated, *“Because when depression is resistant to treatment*,* there is a lot at stake. It could mean losing your social circle*,* losing your job*,* losing your ability to participate in life. These are all things that are also basic needs.”* (IP 11).

Another critical area of concern was the need for protection from self-harm. Several participants reflected on the heightened risk of suicidal thoughts in the context of treatment-resistant depression, and many expressed the need for safeguards to protect service users from themselves during these vulnerable times. One participant expressed, *“And*,* well*,* and that you are protected from yourself during hospital stays*,* because when you’re outside*,* you can’t guarantee anything*,* because you’re so desperate.” (IP 3)*.

In sum, participants described not only the potential risks and benefits of a palliative approach but also detailed the specific forms of support, settings, and safeguards they deem necessary to feel adequately cared for.

## Discussion

This study offers novel insights into how service users with chronic depression understand and evaluate a concept of “palliative psychiatry” and what specific care needs they have that it should address. A central finding is that participants identified current gaps in psychiatric care and interpreted palliative care as something that could potentially bring value to the current psychiatric care system provided that certain preconditions for implementation are met.

The accounts of our participants indicate that a perceived benefit of a palliative approach may lie in reducing the psychological strain associated with repeated unsuccessful treatment attempts. A central element was the mutual acceptance of the chronic nature of the illness by both service users and clinicians leading to a release from cyclical treatment patterns enabling a therapeutic focus on stability and relief. By reframing therapeutic goals toward comfort, stability, and meaningful engagement in life, “palliative psychiatry” might theoretically help break the cycle of false hope and despair and reduce the perceived pressure to achieve full remission. This aligns with arguments from the literature that with each unsuccessful treatment attempt, the likelihood of achieving symptom reduction decreases and somatic and psychological side-effects increase [14].

A potential second positive aspect of a palliative care concept that participants mentioned was the recognition of current suffering and needs. A palliative care perspective may also provide recognition of ongoing suffering and address unmet care needs, particularly regarding continuity of care, low-threshold access, and less institutional, more homelike settings. Several patients also emphasized animal-assisted interventions, underscoring the value of emotional connection and comfort in long-term care. Finally, they expressed wishes for transparent communication and a holistic approach. This aligns with recent ethical commentary emphasizing that futility assessments and care decisions in psychiatry must be grounded in an understanding of the service user as a person with unique values, goals, and life contexts [27]. One example of such a holistic approach is the Oyster Care model developed in Belgium [28]. Importantly, the Oyster Care model introduces the metaphor of an “exoskeleton” to illustrate how a consistent external structure may help sustain individuals with severe, long-standing mental illness. This idea resonates with themes in our interviews, particularly the desire for ongoing support without abandonment and for environments that resemble everyday life.

Alongside the perceived benefits, participants in our study voiced substantial concerns regarding the risks and unintended consequences of a palliative approach. A central concern was the risk of stigmatization, as the term “palliative” was frequently associated with hopelessness and end-of-life care. Clarifying the terminology is therefore essential. The concept of “palliative psychiatry” is not limited to end-of-life care but can be integrated earlier to relieve suffering and reduce harm [14]. Without sensitive framing, “palliative” risks being misread as giving up rather than shifting toward holistic, supportive care. However, misconceptions of terminology do not delegitimize the practical application of palliative concepts.

Another recurring issue related to the handling of suicidality. Some participants explicitly framed the wish for physician-assisted suicide as an expression of autonomy in the face of unbearable suffering and understood this as an important aspect of palliative psychiatry. Conceptually, however, the concept of “palliative psychiatry” strongly differs from assisted dying and, consistent with modern palliative care, intends neither to hasten nor to postpone death [14]. If the concept of “palliative psychiatry” is further explored, an important question will be how to address persistent suicidal wishes in chronically depressed service users. Recent surveys suggest that psychiatrists hold mixed views on physician-assisted suicide in the context of mental illness in general. In a survey among psychiatrists in Switzerland, almost half opposed access to assisted suicide for people with severe and persistent mental illness, while about one-third expressed some degree of support [29]. Further underscoring the cultural dimension, an international survey comparing psychiatrists in India and Switzerland found broad support for palliative psychiatry in both contexts but also notable differences. Indian psychiatrists placed greater emphasis on suicide prevention and were less comfortable with prioritizing quality of life over life expectancy [30].

The results of our study also pointed to systemic and structural barriers. Several participants doubted the feasibility of implementing palliative psychiatry within the current healthcare system, citing constraints such as financial limitations and staffing shortages. Consistent with our participants’ concerns, nurses in residential psychiatry describe structural obstacles such as staffing shortages, administrative load, and limited private spaces that directly constrain the very relational work palliative approaches require [31]. At the same time, other participants in our study mentioned the possible economic viability of a palliative concept compared to repetitive attempts of potentially costly curative treatment cycles. While these points and concerns expressed by service users and raised in the literature must be taken seriously, it remains uncertain whether a palliative approach would ultimately reduce costs or demand additional resources. From an ethical perspective, however, economic considerations represent only one minor aspect and should play a subordinate role in discussing the implementation of palliative psychiatry.

An important consideration concerns the relationship between our sample and the population for which palliative psychiatry has been proposed. While the concept is typically discussed in the context of severe and persistent mental illness, our sample was defined pragmatically based on chronicity and treatment resistance. Importantly, many participants had undergone multiple intensive treatments, including electroconvulsive therapy and ketamine, indicating a high degree of clinical severity. Although our sample may not fully overlap with all patients for whom a palliative approach is considered, it represents a group that is closely aligned with this population. Individuals with long-standing and treatment-resistant depression are often particularly familiar with the limits of curative treatment and were explicitly invited during the interviews to reflect on hypothetical situations in which such limits are reached. Their perspectives therefore provide valuable insights into the needs and concerns that may also be relevant for patients for whom palliative psychiatric approaches are considered. At the same time, more restrictive inclusion criteria might have resulted in a smaller or more ethically complex sample, for example by requiring the inclusion of patients with persistent suicidality. This highlights the practical and ethical challenges inherent in researching this population.

Finally, our study shows that participants worried about the danger of therapeutic nihilism, feelings of abandonment, and premature reorientation of therapy goals. A major challenge contributing to these feelings of abandonment concerns the timing of transition from curative to palliative treatment. Participants in our study feared premature transitions, reflecting broader concerns: unlike oncology, psychiatry lacks biomarkers or prognostic indicators, which heightens the risk of misclassification. Uncertainty about irremediability is also widely discussed in the literature. Ben-Dor et al. [32], for example, emphasize that psychiatry currently lacks reliable prognostic instruments and a shared framework for assessing treatment futility. They call for clearer conceptual and empirical criteria to guide decisions on when further intervention is unlikely to yield meaningful benefit. Moseley [33] argues that judgments of futility in psychiatry inevitably rest on probabilistic and value-laden assessments. Strand et al. [20] emphasize the absence of validated staging criteria and recommend structured, reversible goals-of-care conversations to navigate this uncertainty. Similar debates have arisen in eating disorders, where repeated compulsory feeding without lasting benefit has been framed as medical futility, prompting calls for comfort-focused aims [34]. More recently, a consensus statement by Kious et al. [35] further highlights the conceptual complexities of applying the notion of futility in psychiatry. It emphasizes the challenges of prognostic uncertainty, the risk of premature treatment discontinuation, and the importance of carefully weighing expected benefits and burdens in light of patients’ values.

To address our participants’ concerns, it is important to emphasize that “palliative care” is not intended to connote “giving up” but rather an active reorientation toward achievable outcomes and the relief of suffering [14]. If “palliative” is misunderstood as synonymous with “no further treatment,” service users may feel written off or deprived of care. Such an interpretation would contradict the ethos of palliative care, which emphasizes active, ongoing, and holistic support. Additionally, a palliative treatment plan does not prohibit a return to curative treatment when wished. From the perspective of our participants, a “good” palliative care orientation in psychiatry would be characterized by ongoing, non-abandoning support, transparent yet hope-preserving communication, holistic care, and shared decision-making that respects autonomy while ensuring safety.

### Strengths and limitations

This study has several strengths. To our knowledge, it is the first in-depth qualitative exploration of how service users with chronic depression themselves understand and evaluate the concept of palliative psychiatry. By including a heterogeneous sample in terms of age, gender, and treatment history, we were able to capture a broad spectrum of perspectives. The use of semi-structured, in-depth interviews allowed participants to articulate both personal experiences and hypothetical expectations.

At the same time, several limitations must be acknowledged. First, the study was conducted in a single psychiatric hospital in Germany, which may limit the transferability of findings to other healthcare systems or cultural contexts. Second, although the sample size was appropriate for an in-depth qualitative study and data collection was concluded when no substantially new themes emerged, the overall sample remained relatively small. This may have limited the range of perspectives captured and, consequently, the transferability of the findings. Third, because palliative psychiatry remains largely a conceptual discussion at this stage, participants were asked to respond to a hypothetical scenario, which may not fully reflect how such an approach would be experienced in practice. Fourth, another limitation is the educational homogeneity of our sample, as all participants had at least a high school diploma. This may limit the transferability of our findings to individuals with lower educational attainment, who might have different experiences or needs in psychiatric care. Finally, while our sample reflects a high degree of clinical severity based on treatment history, we did not apply standardized severity measures. In addition, the sample is not fully identical with the patient group for whom palliative psychiatric approaches have been proposed, which should be considered when interpreting the findings.

## Conclusions

The findings of this study have several implications for clinical practice and future research. Future research is needed to further explore the implications of a palliative orientation in psychiatry. In particular, future work should aim to develop criteria to guide decisions about when a palliative orientation may be appropriate, taking into account the uncertainties surrounding prognosis in psychiatry. In addition to that, further qualitative studies involving service users, clinicians and family members could help to better understand how such approaches can be communicated.

Future discussions may explore whether and how conceptual frameworks or guidance could be developed for situations in which a palliative orientation is considered in psychiatric settings. Concrete proposals from the anorexia field such as criteria for a “palliative stage” and formal involvement of palliative medicine and ethics boards might be informative if the field eventually considers developing such protocols [34]. In line with this, Strand et al. [20] have suggested that routine, explicit goals-of-care conversations to align priorities among service users, clinicians, and families could be useful to explore in this context. As palliative psychiatry continues to develop, key questions will include how to communicate its principles to service users, family members and clinicians in a way that prevents fears of stigmatization and feelings of abandonment and how it might theoretically relate to standard psychiatric treatment, not as a terminal pathway but as an additional and reversible therapeutic approach.

Finally, given the strong reservations many participants expressed toward the term “palliative psychiatry”, our findings indicate that further reflection on terminology may be warranted. Language that better conveys the intended aims of relief and continuity – without evoking associations of abandonment or end-of-life care – could facilitate greater acceptance among service users and clinicians. Considering alternative terms or more careful ways of communicating about such care may help reduce misunderstandings and support its thoughtful use in clinical settings.

## Electronic Supplementary Material

Below is the link to the electronic supplementary material.

Supplementary Material 1

## Data Availability

We cannot share research data publicly as individual privacy could be compromised but research data are available from the corresponding author on reasonable request.
