# Supplementary material for: Understanding and evaluation of the concept of “palliative psychiatry” among service users with chronic and treatment-resistant depression: a qualitative study of service user perspectives
Source: BMC Psychiatry. 2026 May 21;26:424. doi: 10.1186/s12888-026-08188-6 (PMC13202979; doi:10.1186/s12888-026-08188-6)
Supplement: Supplementary file 1 — Supplementary Material 1 [file 12888_2026_8188_MOESM1_ESM.docx]

**Supplementary File 1: Summary provided to participants**

**Summary of the concept of “palliative psychiatry” (as provided to participants)**

- Depressive disorders can take a chronic and treatment-resistant course in a substantial proportion of cases.
- It is currently unclear how best to support individuals whose symptoms, such as suicidal thoughts or depressed mood, persist despite treatment.
- One idea is a shift in perspective toward a “palliative” approach.
- Palliative approaches are more commonly used in somatic medicine and are less established in psychiatry.
- In this context, “palliative” does not refer to end-of-life care, but rather to an approach aimed at improving quality of life and alleviating suffering in the context of serious illness.
- It remains unclear what such an approach could look like in psychiatric practice — this is the focus of the interview.

The interview focuses on your personal experiences and ideas; there are no right or wrong answers.
